# A novel multicore Er/Yb co-doped microstructured optical fiber amplifier with peanut-shaped air holes cladding

**DOI:** 10.1515/nanoph-2023-0584

**Published:** 2024-02-16

**Authors:** Yifan Zhang, Yifei Zhao, Ziwei Fang, Jiantao Liu, Changming Xia, Zhiyun Hou, Xuesong Zhao, Zhongwei Tan, Yi Dong, Guiyao Zhou, Jinhui Yuan

**Affiliations:** Guangzhou Key Laboratory for Special Fiber Photonic Devices and Applications, School of Information Optoelectronics Science and Technology, South China Normal University, Guangzhou, Guangdong 510006, China; Key Laboratory of Photonic Information Technology, Ministry of Industry and Information Technology, School of Optics and Photonics, Beijing Institute of Technology, Beijing 100081, China; State Key Laboratory of Information Photonics and Optical Communications, Beijing University of Posts and Telecommunications, Beijing 100876, China

**Keywords:** multicore fibers, microstructured optical fibers, doped fiber amplifiers, space-division multiplexing

## Abstract

The multicore fiber amplifier, as a key component in spatial division multiplexing (SDM) communication systems, presents higher technical difficulty compared to traditional multi-channel single core fiber amplifiers, which has sparked widespread attention. To achieve balance, efficiency, miniaturization, and cost-effectiveness in the performance of multi-core optical fiber amplifiers, we propose an innovative triple cladding 13-core Er/Yb co-doped microstructured fiber (13CEYDMOF). The proposed fiber features an outer cladding with peanut-shaped air holes, which enables uniform excitation of the 13 cores using a single multimode laser pump source within the inner cladding. This approach also prevents damage or aging of the fiber’s outer coating due to the pump laser. Furthermore, the design of Peanut-Shaped Air Holes effectively increases the numerical aperture (NA) of the inner cladding while reducing the outer diameter of the fiber, enhancing the fiber’s mechanical flexibility. To address the coupling difficulties caused by air holes, we bi-directionally pump the 13CEYDMOFA by utilizing a combined technique of the side winding and end pumping. The experimental results show that the 13CEYDMOFA can achieve an average gain of 23.8 dB, a noise figure (NF) of ∼4.6 dB, and an inter-core gain difference of less than 2 dB in the wavelength range of 1529–1565 nm. The in-line amplified transmission experiment demonstrates that the 13CEYDMOFA is well suited for the 13 spatial channels transmission. To the best of our knowledge, this is the first time to realize high performance telecommunication band amplification in a multicore microstructure fiber.

## 1 Introduction

With the exponential growth of global fiber optic network traffic, single-core optical fibers are susceptible to the Shannon limit and may struggle to meet the demands for communication capacity in the future. The SDM technology has emerged as a highly promising solution for achieving large-scale transmission and overcoming the capacity limitations of single-core optical fibers [1], [2], [3]. As a critical component within SDM systems, multi-channel optical fiber amplifiers play a significant role in the application and advancement of future SDM optical communication systems [4], [5]. Compared to single core optical fiber amplifiers, both multicore doped optical fibers and multicore optical amplifier systems present numerous technical challenges. Consequently, many contemporary space-division multiplexing optical communication systems resort to using high-density fiber bundles for multi-channel amplification [6]. This approach not only reduces system integration but also necessitates the deployment of corresponding amplification components for each transmission channel, resulting in high costs. To address these issues, the development of efficient and stable multicore doped optical fibers and amplifiers is urgently required.

The core pumped MCFA can simultaneously provide multi-channel amplifications by attaching a separate pump source to each core [7], [8]. However, it requires the same number of pump channels as the cores, resulting in a complex system. In addition, the restricted core size makes it difficult to deliver the high-power amplification. Recently, the cladding pumped MCFA [9], [10] has emerged as a key technique for reducing the complexity and cost of multi-channel amplifiers, as well as lowering the requirement for pump beam quality. Moreover, it can guarantee the homogeneity of pump light distribution in each core, significantly lessening the inter-core gain difference [11], [12], [13], [14], [15], [16]. In order to couple the pump and signal lights into the MCFA, different kinds of multicore couplers [7], [14] have been used, but they suffer from high insertion loss and low absorption efficiency of the fiber core. Side pumping technique [4], [17], [18], [19] has been shown to be an effective approach for solving the above problems. For the side pumping technique, the pump pigtail is wound on the side of the double cladding MCFA, and a fiber taper is used to couple the pump light into the inner cladding of the MCFA and ultimately into each core. Higher coupling efficiency can be achieved by side pumping technique using *V* groove side coupling [20], embedded mirror side coupling [21], and taper winding [17].

In multicore doped fiber with a cladding pumped method, effectively confining the pump light within the inner cladding and efficiently utilizing it has become crucial. One approach to achieve this goal is to increase the core-to-inner cladding area ratio, thereby enhancing the overlap factor. Following this concept, Chen et al. proposed an annular-cladding 6-core fiber doped with erbium [4]. They achieved this by designing a refractive index distribution within the inner cladding, guiding the pump light into a region with a high refractive index in an annular shape, ultimately entering the fiber core. Building upon a similar principle, an 8-core cladding pumped fiber amplifier with annular erbium doping was designed and fabricated [15]. At present, the low refractive index polymer glue is normally used as the coating layer to construct a double cladding structure. However, this limits the inner cladding numerical aperture (NA), and could lead to some problems, including resistance ability to external mechanical disturbances and cladding glue damage caused by long-term high power pump light leakage. Therefore, we considered an alternative approach by modifying the outer cladding material to enhance the confinement of inner cladding pump light. Microstructured optical fibers (MOFs) have attracted extensive attention in many fields due to their flexible structures [22], [23]. The unique double cladding structure can inhibit the pump light propagation in the inner cladding region [24]. Moreover, it can also reduce the thermal effect loaded on the conventional MCFA through placing the air holes in the cladding region [25]. Despite these advantages, the development of MCMOFA still faces some challenges in meeting the requirements of high gain and low noise amplification at telecommunication wavelengths, as it requires stringent specifications for gain materials, core size and number, and fiber structure. In addition, the air hole isolation band used in MCMOFA prevents the usage of the conventional side pumping method.

In this paper, to address these challenges, we propose and fabricate the 13CEYDMOFA for multi-channel amplifications at telecommunication wavelengths. Our approach involves arranging a circle of air holes as the isolation layer to form a triple cladding structure, which can increase the NA of the inner cladding and the load capability for high pump power. We also develop an Er/Yb co-doped rare earth material using a non-chemical vapor deposition (NCVD) technique [26] to increase the concentration rate of rare earth materials, improve energy conversion efficiency, and enhance the amplification effect. To solve the coupling problem of the MCMOFA, we use a combined method of the side winding and end pumping (SWEP). Under the condition of all-fiber bidirectional pumping, the proposed 13CEYDMOFA achieves an average gain of 23.8 dB, a NF of ∼4.6 dB, and an inter-core gain difference of less than 2 dB in the wavelength range of 1529–1565 nm. Finally, we integrate it into an all-fiber 13-channel transmission link and confirm its suitability for an in-line amplified SDM system.

## 2 Design and fabrication of the 13CEYDMOFA

**Figure 1:**
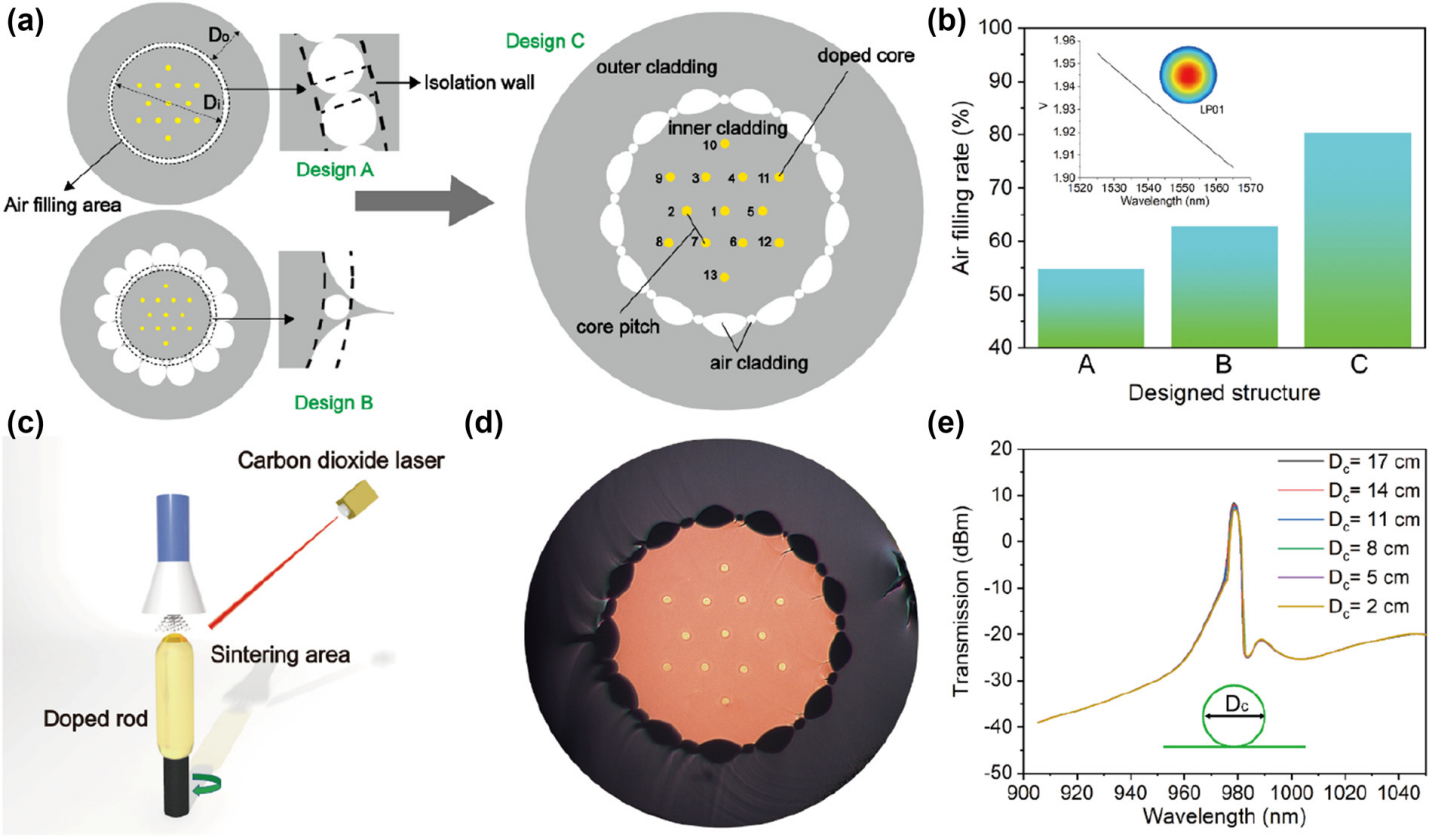
Design and fabricaion of the 13CEYDMOF. (a) Cross-sectional structure of the designed 13CEYDMOF with different air holes claddings. (b) Air filling rate for all the triple cladding structures and the calculated normalized frequency *V* of each core of the 13CEYDMOF. (c) Laser powder sintering method for preparing the core rod. (d) Cross-section of the fabricated 13CEYDMOF. (e) Transmission spectra of the inner cladding layer of the 13CEYDMOF at different bending rates.

In microstructured optical fibers, multiple air-hole layers can enhance the confinement of the inner-layer light. However, as the number of fiber cores increases, the presence of multiple layers of air holes unavoidably leads to an excessively large overall fiber diameter, which is unfavourable for pump coupling and connection with passive devices. Therefore, the construction of a dual-cladding structure with a single layer of air holes (Design A), as depicted in Figure 1(a), is prioritized. According to the numerical aperture calculation formula $NA=\sqrt{n_{1}^{2}-n_{2}^{2}}$ the refractive index *n*_1_ of the inner cladding is the refractive index of quartz. Reducing the average refractive index *n*_2_ of the second cladding layer can result in an increased NA. The isolation wall, which is determined by the quartz wall between two adjacent air holes directly affects the air filling rate of the second cladding. One possible method is to reduce the number of isolation walls by increasing the diameter of the air holes, but large air holes can cause the quartz wall to thicken. Consequently, we endeavour to consolidate multiple closely positioned air holes into larger ones and fill the intervening spaces with smaller holes (Design B). This approach can effectively enhance the air filling factor of the cladding layer by reducing the number of isolation walls between air holes. The number of isolation walls in Design B, relative to Design A, has decreased from 72 layers to 13 layers. Nevertheless, while ensuring the constant diameter of the inner cladding (Di) and the outer silica layer (Do), the inclusion of large circular holes increases the fiber diameter. To address this issue, without changing the number of isolation walls, we compress the merged air holes into elliptical shapes. Due to the limited contact area of elliptical holes, this not only reduces the air-filling ratio but also leads to poor stability of the drawing process. As a remedy, we introduce smaller air holes between the elliptical holes, constructing a peanut-shaped cladding structure for the multicore optical fiber (Design C).

The air filling rate for each designed structures are then calculated, and the results are shown in Figure 1(b). It is obvious that the structure of Design C has the highest air filling rate of 80.42 % compared to Design A of 54.83 % and Design B of 62.79 %. Therefore, we choose Design C as the final structure. The calculated normalized frequency *V* of each core is depicted in the insect of Figure 1(b), where *V* < 2 implies that the fiber supports the multi-channel single-mode transmission in the wavelength range of 1525–1565 nm.

To prepare the Er/Yb co-doped core rod, the laser powder sintering method is utilized, as depicted in Figure 1(c). By using a high-power laser to melt the doped powder and silica material, we can precisely control the concentration and proportion of various dopants by adjusting the powder discharge speed and the moving speed of the preform rod. This allows us to further improve the doping concentration ratio. Then the doped glass rod is etched and polished to the designed size. For the conventional MOF, the stack-and-draw method is preferred when preparing a preformed rod. But for the MCMOF, it is challenging to maintain the spatial balance, particularly the distance between the fiber cores by using the conventional stack-and-draw method. This would result in variable insertion loss and unequal gain in a MCMOFA. To address this problem, we introduce a novel multilayer stack method that uses the quartz sleeves and capillary rods to stabilize each layer of preform. Firstly, due to the different coefficients of thermal expansion between the doped material and quartz, directly stacking the fiber cores and quartz rods may result in irregular shapes of the fiber cores, thereby affecting the spatial distribution of the cores. Therefore, we place the coarse material rod fabricated in Figure 1(c) into a quartz sleeve of the corresponding size for the first pre-drawing, forming a uniformly sized core rod surrounded by quartz, which is then cut into 13 core rods of completely consistent dimensions. Next, a method similar to stack-and-draw method is applied to prepare the preform rods for the inner cladding. Subsequently, the preform rods for the inner cladding were placed in a thicker quartz sleeve for filling and solidification. Another layer of wide quartz tube is then added outside the quartz sleeve, and between these two layers of sleeves, multiple layers of small-sized quartz rods with uniform dimensions are arranged. Hollow tubes of the designed dimensions are used to replace the small-sized quartz rods, forming a peanut-shaped air cladding. Finer quartz rods are inserted into the gaps left by the replacement to stabilize the structure. The final drawing of the multi-core microstructure optical fiber was completed in the laboratory’s drawing tower. It maintains the structural symmetry and core-to-core distance accurately during the high-temperature drawing process. The stacking of multi-layer quartz sleeves ensures that the spatial position of the fiber core remains steady. As a result, the difference in each core size is less than 0.1 μm.

The cross-section of the fabricated 13CEYDMOF is shown in Figure 1(d). Based on an electron microscope test, the diameter of the 13 cores distributed in the inner cladding region is 7.2 μm, the average distance between the neighbor cores is 42 μm, the diameter of the inner cladding region is 240.8 μm, and the most outer cladding region is 437.8 μm which is coated with normal polymer glue. The refractive index difference (Δ) between the core and inner cladding region has been tested to be 0.41 % at a wavelength of 1550 nm, and the NA of the active fiber core is 0.13.

To validate the confinement capability of the designed peanut-shaped dual-cladding optical fiber for pump light, we subsequently conducted experimental tests on the 13CEYDMOF subjected to external perturbations. The light from a 976 nm multimode laser is coupled into the13CEYDMOF, and the fiber is bent with different diameters (Dc). As shown in Figure 1(e), when Dc is varied from 17 to 2 cm, no prominent degradations in the transmission spectra are observed. This indicates that the proposed 13CEYDMOF has high resistance to the external perturbations due to the surrounding air holes.

## 3 Experimental results

### 3.1 Pumping and coupling devices for the 13CEYDMOF

**Figure 2:**
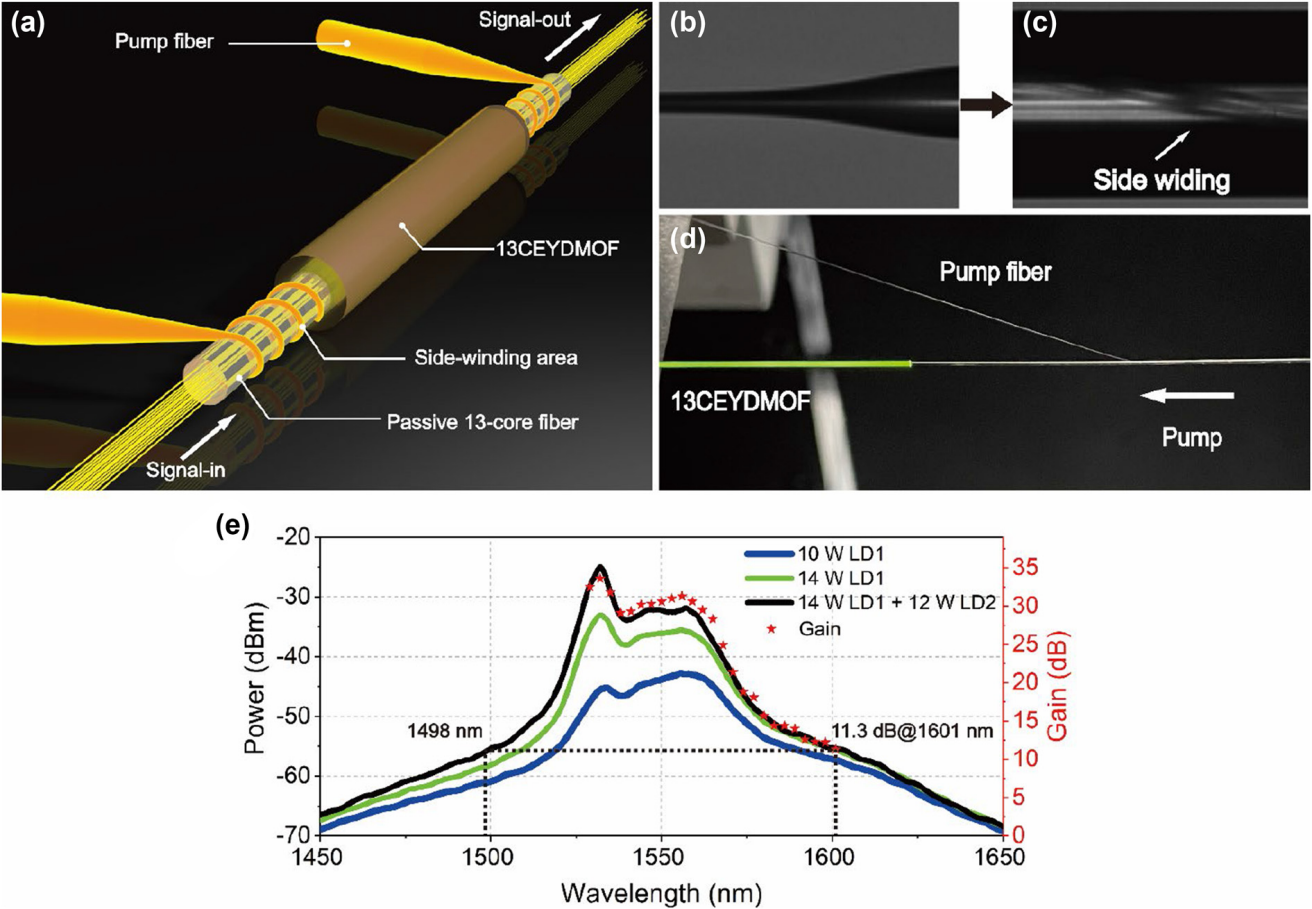
Preparation and experiment verification of the SWEP method. (a) Schematics of the SWEP method. (b) Optical image of the tapered pump fiber. (c) Passive 13-core fiber wound by the tapered pump fiber. (d) Photograph of one coupling device. (e) The ASE spectra of the 13CEYDMOFA with different pump powers and the measured gain spectrum of one core.

The SWEP method is used to prepare the coupling device, as shown in Figure 2(a). Owing to a high isolation characteristic of the air holes distributed in the second layer of the 13CEYDMOFA, conventional side-pumping techniques are ineffective in coupling pump light into the inner cladding region. One possible approach is to couple the signal and pump lights together before entering the 13CEYDMOFA. First, the pump pigtail fiber is tapered by a multifunctional fiber fusion, as shown in Figure 2(b). To ensure the coupling efficiency, the lengths of the front-end transition region, the rear end transition region, and the middle region are set as 5 mm, 15 mm, and 20 mm, respectively, and the diameter of the tapered region is 20 μm. The tapered pump fiber is then wound on the side of the passive13-core fiber, as shown in Figure 2(c). The core of the 13-core passive fiber has a trapezoidal refractive index distribution, where the diameter of the reflective index flat area is 7.6 µm and the diameter of the reflective index gradient area is 2.2 µm. The core distance is 42 µm to match the active fiber. Second, two sections of MCFs are spliced by using a carbon dioxide laser fusion splicer. The fusion losses for the 13 fiber cores are consistently maintained below 0.5 dB. In the subsequent gain tests, these insertion losses are not subtracted, aligning more closely with engineering realities. The prepared multi-core MOF pump coupling end is shown in Figure 2(d) after loading the multimode pump light at 976 nm. The green light on the left 13CEYDMOFA is caused by up-conversion photoluminescence. Unlike the fusion process of ordinary MCF, the sufficient laser power needs to be provided in the fusing process to ensure the coupling efficiency. In the meantime, the air holes of the 13CEYDMOFA cannot be collapsed to minimize the leakage of pump light at the fusing point especially when the laser power is very high. Overall, this method allows for simultaneously coupling the signal and pump lights into the 13CEYDMOFA.

To verify the reliability of the special SWEP method, we measure the amplified spontaneous emission (ASE) spectrum, as shown in Figure 2(e). As the forward pump (LD1) power increases from 10 to 14 W, the intensity of ASE gradually becomes stronger. Subsequently, the ASE strength is further increased when the backward pump (LD2) is loaded. For the conventional Er/Yb co-doped phosphorus silicate fibers, the gain effect at the shorter wavelength side is poor due to the changes in the absorption/emission cross-section [27]. But for the proposed 13CEYDMOFA, the peak gain is observed at wavelength 1532 nm from the ASE spectra, and the gain also maintains a high level at the shorter wavelength side. This is mainly because only a section of 2.1-m-long gain fiber is required, and the shorter gain fiber makes the peak gain wavelength of the ASE spectrum emerge at shorter wavelength side [18]. Therefore, by choosing a signal wavelength close to the peak wavelength, a higher gain can be obtained. To explore the potential of the 13CEYDMOFA in terms of bandwidth, we also measure the gain spectrum of one core in the wavelength range from 1529 to 1601 nm. As shown in Figure 2(e), the gain spectrum closely resembles the ASE spectrum under the same pump condition. At wavelength 1601 nm, the gain reaches 11.3 dB, while the corresponding ASE intensity at the short wavelength side is at 1498 nm. Thanks to the unique structure and material preparation method, the proposed 13CEYDMOFA has the potential to achieve the broadband amplification in the telecommunication band.

### 3.2 Optical characteristics and amplification performances of the 13CEYDMOFA

To characterize the optical absorption and transmission properties of the 13CEYDMOFA, we use a cut-back method to test its absorption. The pump pigtail fiber is fused with the end face of the 13CEYDMOFA, the gain fiber is cut off, and the output power is recorded. Besides, we use a broadband light source (NKT Photonics) as the input signal for the 13CEYDMOFA and measure the background loss of the gain fiber. The cladding absorption is 9.16 dB/m at 976 nm, and the average background loss is 0.03 dB/m in the wavelength range from 1100 to 1300 nm, without affecting the amplification performances.

The amplification performances of the 13CEYDMOFA are then measured using the SWEP method shown in Figure 2(a). The signal light power is controlled by an optical fiber attenuator before entering the optical spectrum analyzer (OSA). By adjusting the optical fiber attenuator, the signal light power is recorded as the Signal-in. Next, the adjusted signal port is connected to a Fan-in device, and the multimode pump light at 976 nm and signal light are coupled into the tail fiber and gain fiber by the SWEP method. Fan-in and Fan-out devices are prepared using the identical 13-core passive fiber as the tail end, ensuring that the insertion loss for each core remains below 0.3 dB to meet the transmission requirements. After the signal is amplified by the 13CEYDMOFA, its output part is fused with a Fan-out device. The bi-directional pumping is applied, and the pigtail fiber is simultaneously SWEP-connected to both forward and backward ends of the gain fiber. The 13-core fiber is coated with high refractive index glue, enabling it to act as the transmission part as well as a pump striping device. The output signal is recorded as the Signal-out, and the amplification performances can be characterized by the OSA.

**Figure 3:**
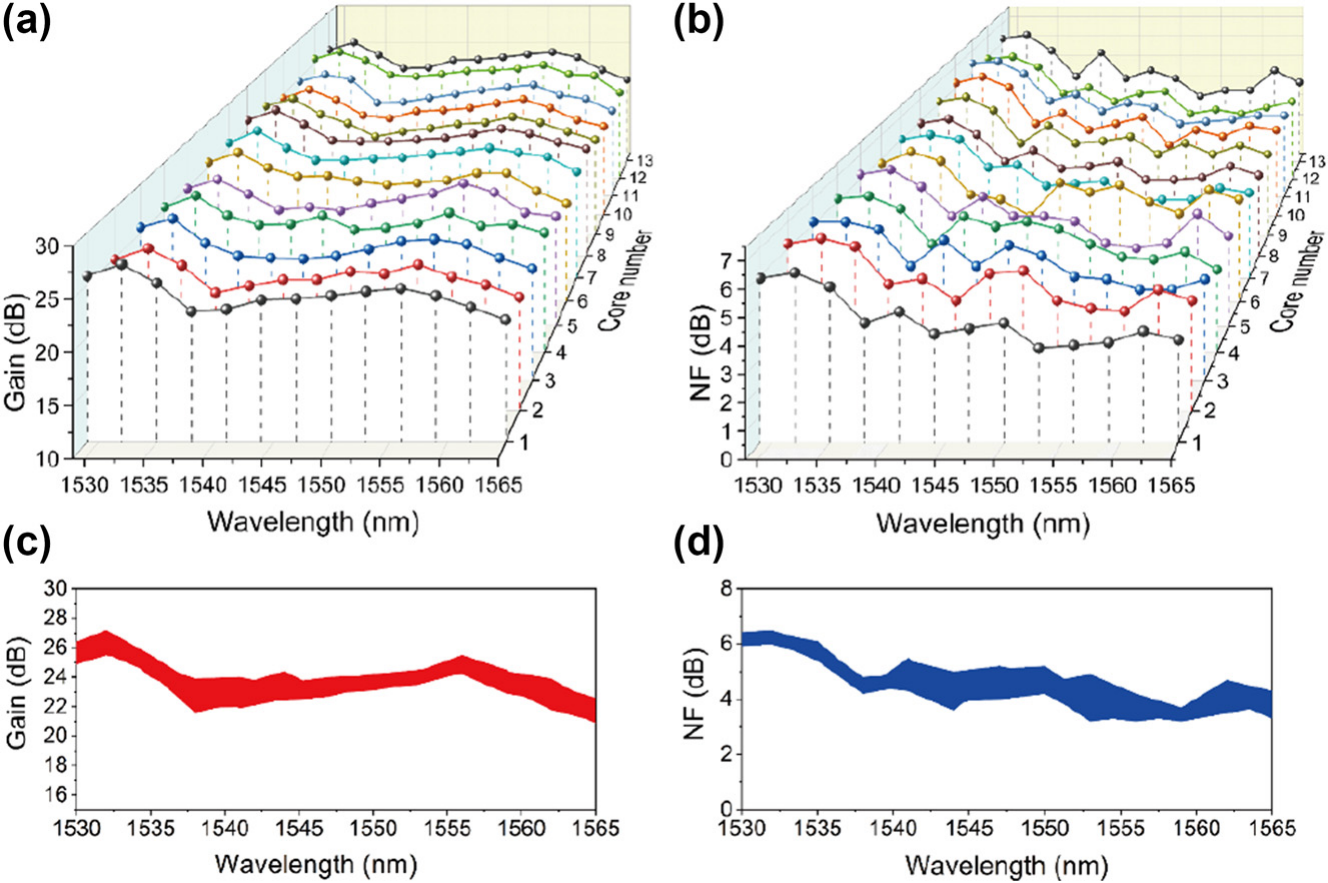
Amplification performance of 13CEYDMOFA at different wavelengths. The measured (a) gain and (b) NF spectra for all 13 cores of the 13CEYDMOFA for an input signal power of −4 dB m when both the forward and backward pumping are loaded. (c) Gain and (d) NF ranges for all 13 cores of the 13CEYDMOFA.

Figure 3(a) and (b) show the measured gain and NF spectra for all 13 cores of the 13CEYDMOFA when the forward pump power is 14 W, backward pump power is 12 W, and input signal power is −4 dB m. In the wavelength range of 1529–1565 nm, the proposed 13CEYDMOFA achieves an average gain of 23.8 dB and an average NF of 4.6 dB. The maximum gain of 27.2 dB is obtained at wavelength 1532 nm. In addition, the NF remains below 5 dB at longer wavelength side and higher at shorter wavelength side. This is mainly due to the reductions in the reabsorption and particle number inversion of the 13CEYDMOFA. Figure 3(c) and (d) show the fluctuation ranges of the gain and NF for all 13 cores of the 13CEYDMOFA. The inter-core gain difference between different cores is less than 2 dB, which indicates that the proposed 13CEYDMOFA has uniform geometric dimension and Er/Yb doping concentration for each core.

**Figure 4:**
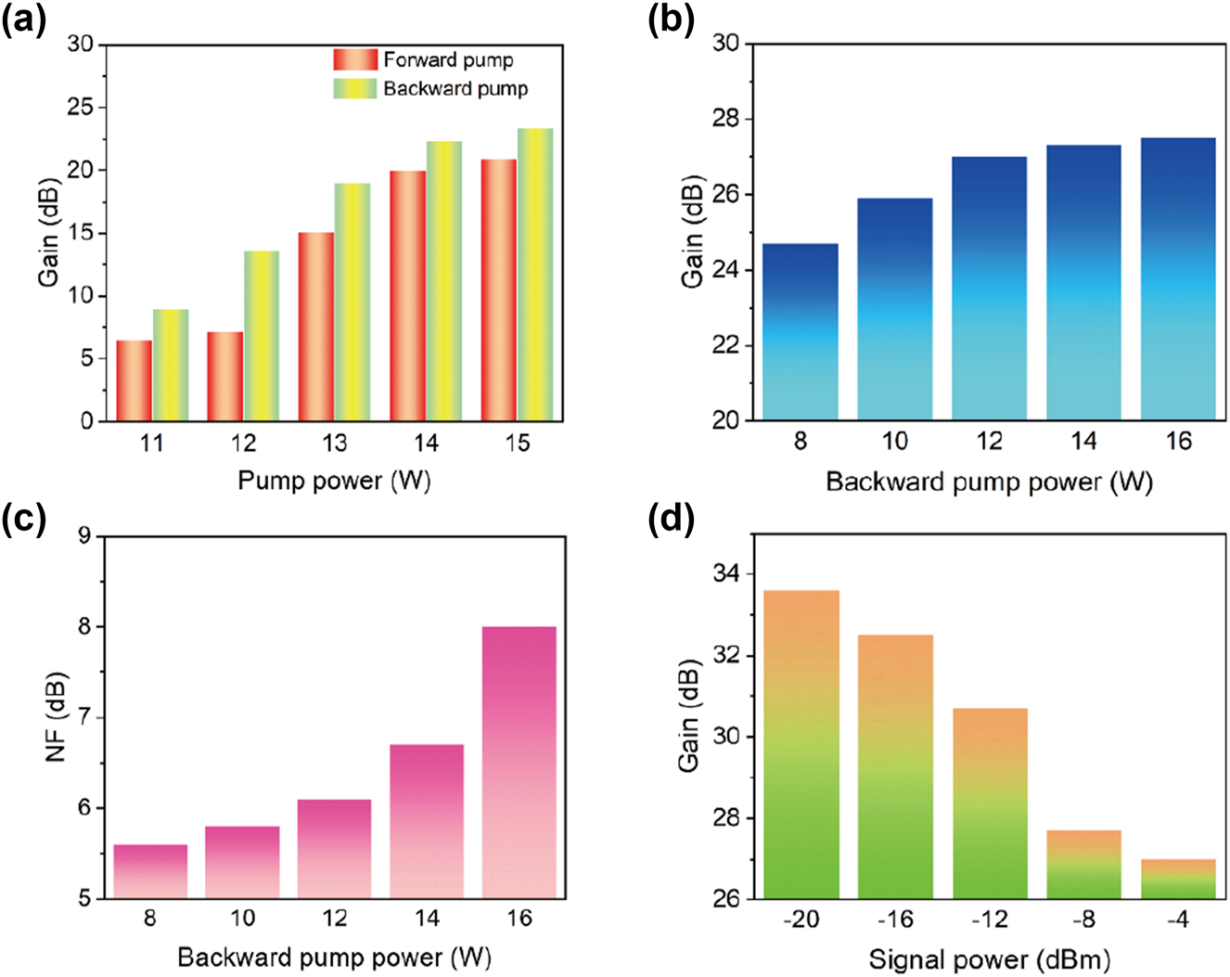
Amplification performance of 13CEYDMOFA under different pump and signal conditions. (a) Gain of the core 1under different forward or backward pump powers. (b) Gain and (c) NF of the core 1 when the forward pump power remains 14 W and the backward pump power is varied from 8 to 10, to 12, to 14, and to 16 W, respectively. (d) Gain of the core 1 under different signal powers.

By adjusting the pump and signal powers, we investigate the gain and NF performances of the 13CEYDMOFA. In Figure 4(a), the gain of core 1 is depicted with an input signal power set at −4 dB m for each wavelength. The forward and backward pump power is varied from 11 to 12, 13, 14, and 15 W, respectively. When the forward pump power reaches 14 W, the gain value is higher than 20 dB, and the rising trend becomes relatively flat due to the pump gain saturation. For the backward pump, the gain value is always higher than that of the forward pump, and it also tends to be flat as the pump power increases. Figure 4(b) and (c) show the gain and NF of the core 1 when the forward pump power remains 14 W and the backward pump power is varied from 8 to 10, to 12, to 14, and to 16 W, respectively. When the backward pump power is 12 W, the maximum gain value is up to 27 dB. At this point, the 13CEYDMOFA is close to the gain saturation, and the gain growth trend becomes flat. However, the NF also increases significantly.

To balance the gain and NF, we ultimately select a forward pump power of 14 W and a backward pump power of 12 W. In Figure 4(d), the gain of the core 1 under different signal powers is also investigated. When the power of signal light at wavelength 1532 nm is changed from −20 to −4 dB m, the gain value gradually decreases. The gain reaches the maximum value of 33.6 dB with the signal power of −20 dB m. When the pump power remains constant, a higher gain value can be obtained at a lower signal power.

**Figure 5:**
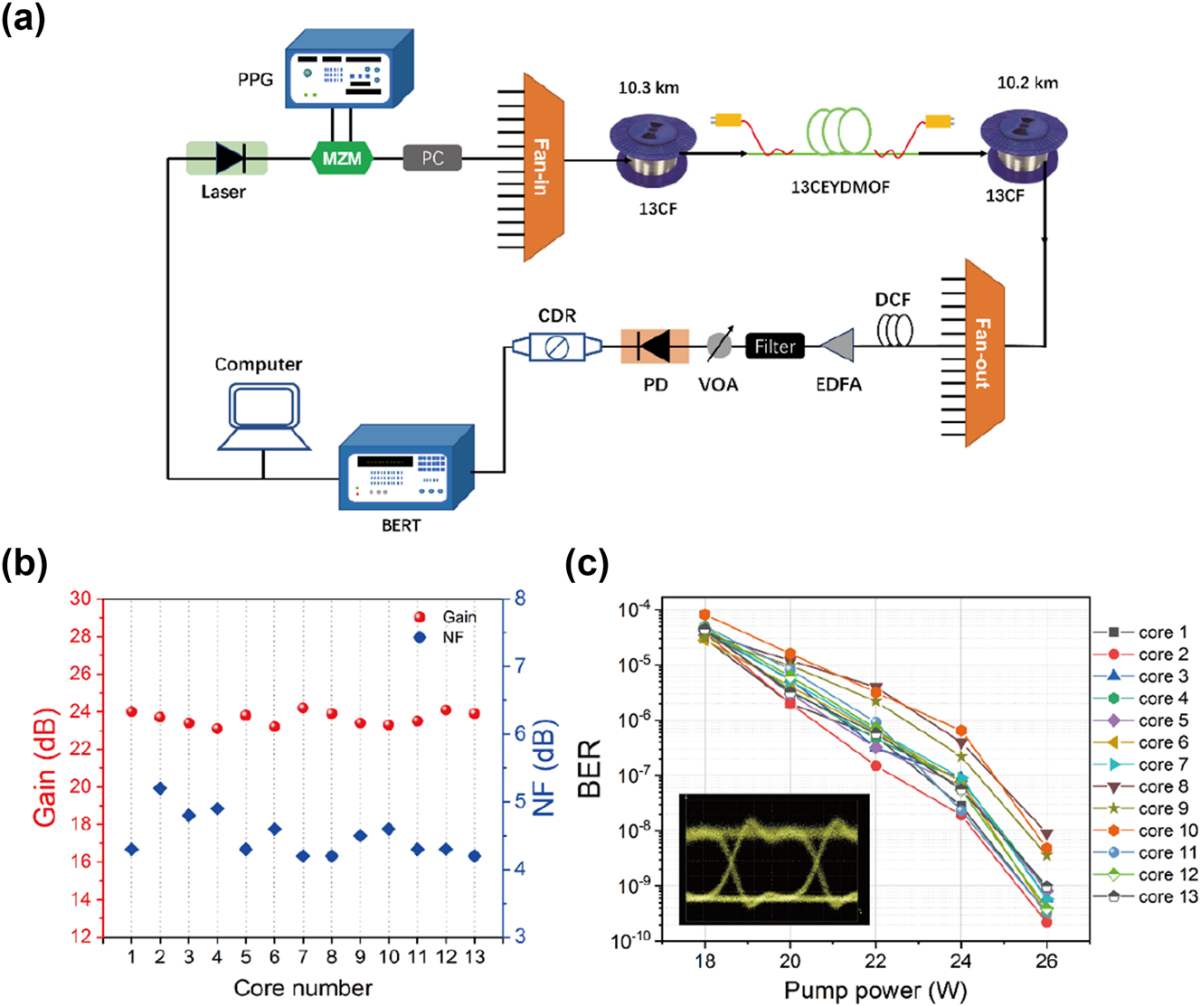
Multi-channel transmission experiment. (a) The experimental setup for an in-line SDM transmission link. PPG, pulse pattern generator; MZM, Mach–Zehnder modulator; PC, polarization controller; DCF, dispersion compensation fiber; EDFA, erbium doped fiber amplifier; VOA, variable optical attenuator; PD, photodetector; CDR, clock data recovery; BERT, bit error rate tester. (b) Gain and NF for all 13 cores at signal wavelength 1550 nm. (c) The measured BERs for different cores when the forward pump power remains 14 W and the backward pump power is varied from 4 to 6, to 8, to 10, and to 12 W, respectively. The inset of (c) shows the eye diagram of the transmission in the core 1.

The experimental setup for an in-line 13 spatial channels transmission link is shown in Figure 5(a). In the transmitter, the 10-Gbit/s on-off keying (OOK) data signals are generated by a pulse pattern generator (PPG) and used to modulate the CW light by a Mach–Zehnder modulator (MZM). The all-fiber SDM link comprises two sections of 13-core transmission fibers, each with lengths of 10.3 km and 10.2 km. A bidirectionally pumped 13CEYDMOFA serves as the relay amplifier section. At the output end of the Fan-out, a dispersion compensation fiber (DCF) is connected to eliminate the effect of chromatic dispersion. An erbium-doped fiber amplifier (EDFA) is inserted to compensate for the losses caused by the components in the transmission link. The transmitted signals are attenuated by a variable optical attenuator (VOA) and measured by a photodetector (PD). The clock data recovery (CDR) module is used for the clock recovery and data decision, and a bit error rate tester (BERT) is used for measuring the bit error rate (BER). The 13-core transmission fibers have low transmission loss of 0.5 dB/km and small inter-core crosstalk of less than −50 dB/km for all cores.

In the experiment, a signal light at wavelength 1550 nm is loaded into different fiber cores for transmission. First, when the forward pump power is 14 W and backward pump power is 12 W, we measure the gain and NF of the signal light for all 13 cores, as shown in Figure 5(b). In Figure 5(c), the BERs of all 13 cores are tested when the forward pump power remains 14 W and the backward pump power is varied from 4 to 6, to 8, to 10, and to 12 W, respectively. As the total pump power increases from 18 to 26 W, the gain value of each core increases, and the corresponding BER decreases to below 10–9, which confirms that the proposed 13CEYDMOFA is well suited for the SDM transmission. In addition, it is found that the BERs of some cores are relatively higher than those of the other cores. This is due to the different dispersion effects and transmission losses for all 13 cores during the long-distance transmission. Moreover, the eye diagram of the transmission in the core 1 is shown in the inset of Figure 5(c). It can be known from Figure 5(c) that the multi-channel transmission performances including the BER and eye diagram could be obviously improved by further optimizing the geometric structure parameters of the 13CEYDMOFA.

## 4 Conclusions

In summary, we propose and fabricate a novel 13CEYDMOFA with peanut-shaped triple cladding structure for multi-channel amplifications at telecommunication wavelengths. The unique structure of the air holes allows for uniform pumping of the inner cores using a single laser source, greatly enhancing system integration. By replacing the existing low refractive index coating with the air holes combined with quartz cladding, the pump laser’s damage to the cladding is effectively prevented, thereby increasing the device’s lifespan. Additionally, the high refractive index difference between the air cladding and quartz enables a larger NA for the inner cladding, addressing the issue of weak pump light confinement. We utilize the peanut-shaped air holes structure to improve the air-filling factor, which not only enhances the NA but also reduces the fiber size, thereby enhancing its mechanical flexibility. The SWEP method is used to solve the coupling problem of the 13CEYDMOFA. When the input signal power is −4 dB m, the 13CEYDMOFA can achieve an average gain of 23.8 dB, a NF of ∼4.6 dB, and an inter-core gain difference of less than 2 dB in the wavelength range of 1529–1565 nm. Finally, it is demonstrated that the proposed 13CEYDMOFA can be well applied for an in-line long-distance multi-channel transmission. As a new type of multi-channel optical fiber amplifier, the proposed 13CEYDMOFA is expected to have important application in the next generation SDM system.
